# Rapid Molecular Assays for Specific Detection and Quantitation of *Loa loa* Microfilaremia

**DOI:** 10.1371/journal.pntd.0001299

**Published:** 2011-08-30

**Authors:** Doran L. Fink, Joseph Kamgno, Thomas B. Nutman

**Affiliations:** 1 Laboratory of Parasitic Diseases, National Institute of Allergy and Infectious Diseases, National Institutes of Health, Bethesda, Maryland, United States of America; 2 Filariasis Research Centre and Faculty of Medicine and Biomedical Sciences, University of Yaounde I, Yaounde, Cameroon; Washington University School of Medicine, United States of America

## Abstract

**Background:**

Accurate diagnosis of *Loa loa* infection is essential to the success of mass drug administration efforts to eliminate onchocerciasis and lymphatic filariasis, due to the risk of fatal encephalopathic reactions to ivermectin occurring among highly microfilaremic *Loa-*infected individuals living in areas co-endemic for multiple filarial species.

**Methodology/Principal Findings:**

From a pool of over 1,800 *L. loa* microfilaria (mf) expressed sequence tags, 18 candidate *L. loa* mf-specific PCR targets were identified. Real-time PCR (qPCR) assays were developed for two targets (LLMF72 and LLMF269). The qPCR assays were highly specific for *L. loa* compared with related filariae and also highly sensitive, with detection limits of 0.1 pg genomic DNA, or 1% of DNA extracted from normal blood spiked with a single *L. loa* microfilaria. Using various DNA extraction methods with dried blood spots obtained from Cameroonian subjects with parasitologically proven loiasis, the LLMF72 qPCR assay successfully estimated mf burden in 65 of 68 samples (50–96,000 mf/mL by microscopy), including all 12 samples subjected to a simple 10-minute boiling extraction. Additionally, the assay detected low-level microfilaremia among 5 of 16 samples from patients thought to be amicrofilaremic by microscopy.

**Conclusions/Significance:**

This novel, rapid, highly sensitive and specific qPCR assay is an important step forward in the laboratory diagnosis of *L. loa* infection.

## Author Summary

*Loa loa* is a filarial nematode that infects over 10 million people in Africa. Most infections cause no symptoms, but individuals with large numbers of blood-stage microfilariae are at risk for fatal reactions to ivermectin, an antiparasitic agent used to treat and prevent infections with *Onchocerca volvulus*, a related filarial parasite that may occur alongside *L. loa*. To address the urgent need for a point-of-care *L. loa* diagnostic assay, we screened a *Loa* microfilaria gene expression library and identified 18 *Loa*-specific DNA targets. From two targets, we developed a novel, rapid quantitative PCR assay for estimating *L. loa* microfilaria burden. The assay is highly sensitive (detects a single microfilaria in 20 µL of blood) and correlates well with microfilaria counts obtained with conventional microscopic techniques. The assay is species-specific for *L. loa* compared with related filarial parasites (including *O. volvulus*) and can be used in its current form in resource-rich areas as a diagnostic tool for *L. loa* infection. Although modifications will be required to make point-of-care use feasible, our assay provides a proof of concept for a potentially valuable tool to identify individuals at risk for adverse reactions to ivermectin and to facilitate the implementation of filarial control programs.

## Introduction

*Loa loa* is a filarial nematode that infects between 3 and 13 million people in Central and Western Africa. Although infected individuals may exhibit a range of symptoms, indigenous inhabitants of endemic areas are typically clinically asymptomatic even in the setting of high parasite burden. Loiasis is nonetheless an important public health concern due to the occurrence of greater than 1,000 severe adverse reactions (including fatal encephalopathies) in *Loa*-infected individuals receiving ivermectin through mass drug administration (MDA) programs aimed at the elimination of onchocerciasis and lymphatic filariasis. Consequently, disruption of MDA has occurred in certain communities where these filarial diseases are co-endemic [1], [2].

The mechanism of *Loa*-related post-ivermectin encephalopathy is unclear but risk appears to be greatest with microfilaremia above 8,000 organisms/mL [3], [4]. Preventing disruption of MDA therefore necessitates identification of persons with high-level *L. loa* microfilaremia. Microscopic examination of mid-day blood samples is currently the only diagnostic method routinely used in endemic areas. Due to the need for examiner expertise in parasite morphology and the effort required to process large numbers of samples, this method is impractical for use as a widespread screening tool.

Several alternative methods for the definitive diagnosis of loiasis have been evaluated in research settings, but none is used clinically in endemic areas. Most *Loa-*infected individuals will exhibit a positive IgG antibody response to crude protein extracts of *Brugia malayi*, but this assay is cross-reactive among all filarial pathogens as well as some intestinal helminthes [5]. Newer serologic assays based on the *L. loa* SXP-1 antigen and others reliably distinguish loiasis from other filarial and helminthic infections but cannot discern between active and prior infection or quantify microfilaremia [6], [7]. *Loa-*specific conventional polymerase chain reaction (PCR) assays have been developed, but these are time-consuming, not quantitative, and not generally available for use in clinical settings [8]–[12].

Molecular methods for quantitative detection of parasitic agents [13]–[16] require selection of appropriate target sequences. We therefore sought to identify *L. loa* molecular target sequences suitable for incorporation into a rapid, sensitive, and high-throughput quantitative PCR assay that might allow reliable, species-specific determination of *L. loa* microfilaremia without the need for formal training in conventional parasitologic methods. To this end, we used novel bioinformatics strategies to parse large numbers of *B. malayi*, *Wuchereria bancrofti*, *Onchocerca volvulus*, and *L. loa* transcripts based on expressed sequence tags (ESTs). We report here a set of *L. loa* microfilaria (mf)-specific target sequences with incorporation of two into real-time PCR assays that allow for enumeration of microfilaremia.

## Methods

### Patient samples

All samples were acquired under registered protocols approved by the Institutional Review Board of NIAID (NCT00001230), the Cameroon Ethical Committee and the Cameroon Ministry of Health with written informed consent obtained from all subjects.

### Filarial organisms

*L. loa, W. bancrofti*, and *Mansonella perstans*: mf were purified from the blood of patients seen by the NIH/NIAID Clinical Parasitology Unit. *O. volvulus*: adult worms were obtained from excised onchocercomas of Guatemalan patients. *B. malayi*: mf and adult worms were obtained from the Filariasis Research Reagent Repository Center (Athens, GA, USA).

### Extraction of genomic DNA

*L. loa* (5 million mf) and *O. volvulus* (50 adult female worms) were digested overnight at 56°C in buffer G2 (Qiagen) with 20 mg/mL proteinase K. Genomic DNA was extracted using Genomic tip-100/G and Genomic DNA buffer set (Qiagen, Valencia, CA, USA). The remaining filarial organisms were digested as described above, and genomic DNA was extracted with phenol/chloroform.

### Extraction of mf RNA

One million *L. loa* mf and 500,000 *B. malayi* mf were frozen under liquid nitrogen and disrupted by a stainless steel piston/mortar apparatus. Total RNA was extracted using the RNeasy Kit (Qiagen, Valencia, CA, USA), and poly-A RNA was isolated with the Oligotex mRNA Mini Kit (Qiagen, Valencia, CA, USA).

### Synthesis of mf cDNA

cDNA was synthesized from 1 µg of *L. loa* or *B. malayi* total RNA in 50 µL reactions containing 160 units MultiScribe reverse transcriptase, 5.5 mM MgCl_2_, 2mM dNTP mix, 2.5 mM random hexamers, 20 units RNAse inhibitor, and 1X RT buffer (Applied Biosystems, Foster City, CA, USA).

### Construction and screening of a *L. loa* mf cDNA library

A cDNA library was created in pTriplEx2 using the SMART cDNA Library Construction Kit (Clontech, Mountain View, CA, USA). The library was screened by PCR amplification of individual plaques using primers specific to the pTriplEx2 phagemid insertion site (table 1) and sequencing at the NIAID Rocky Mountain Laboratories Genomics Unit (Hamilton, MT, USA).

**Table 1:** Primer and probe sequences for PCR assays.

Assay	Sequence	Product size (bp)
**RT-PCR**		
LLMF72 5′ primer	5′-GAGGGATCCATGCGGCATTCCTCTATAAAC-3′	252
LLMF72 3′ primer	5′-GAGCTCGAGTCAACTCCCTTTGAAACGTTT-3′	
LLMF269 5′ primer	5′-GAGGGATCCATGAAAGCTGTTGGTGCCATT-3′	398
LLMF269 3′ primer	5′-GAGCTCGAGTCACAACCTTTCGTTCTTCAT-3′	
**qPCR**		
LLMF72 5′ primer	5′-CGGAAGACTCAACGTCAGAAATCA-3′	62
LLMF72 3′ primer	5′-AGGAACGCTTGATGGTGATGT-3′	
LLMF72 probe	5′-FAM-CCAACAGCCTGCTTTT-NFQ-3′	
LLMF269 5′ primer	5′-GCAAGAGTCTTTACAACTATATTTTGCGAAA-3′	78
LLMF269 3′ primer	5′-GGCATCTTCATCCGGGTAACTATAC-3′	
LLMF269 probe	5′-FAM-TCGAGACGAGACTTTC-NFQ-3′	

### Contig construction and bioinformatics analysis

*L. loa* mf ESTs were assembled into contigs using the Desktop cDNA Annotation System (dCAS 1.4.3) software package [17]. Contigs were selected for further evaluation as candidate assay targets based on the number of ESTs comprising the contig (abundance), length of at least 200 bp with a predicted open reading frame (ORF), and lack of sequence homology to *i)* the non-redundant protein database (nr), *ii)* ESTs from related filarial pathogens, and *iii) L. loa* L3 larval stage ESTs (D. L. Fink et al., unpublished).

### Reverse transcriptase (RT)-PCR of candidate target transcripts

RT-PCR was performed on *L. loa* total RNA using the OneStep RT-PCR kit (Qiagen, Valencia, CA, USA) and primers specific to the 5′ and 3′ ends of each target transcript (table 1). Quantification of specific PCR product was accomplished using a 2100 Bioanalyzer instrument and 2100 Expert software (Agilent Technologies, Waldbronn, Germany).

### DNA extraction from whole blood spiked with *L. loa* mf

*L. loa* mf were spiked into 200 µL aliquots of whole blood obtained from a healthy volunteer with no history of exposure to filaria-endemic regions. Following zinc BB disruption [18], DNA was extracted using the QiaAmp DNA blood and kit (Qiagen, Valencia, CA, USA). Duplicate sets of spiked whole blood samples were created by adding intact *L. loa* mf as described above to 50 µL aliquots of whole blood. After addition of 150 µL distilled water, the samples were vortexed briefly then boiled for 10-30 minutes. Following removal of 2 µL aliquots, samples were spun in a bench-top centrifuge at maximum speed for 5 minutes and supernatants recovered.

### Blood spot collection

Mid-day venous blood samples were obtained by fingerprick from Cameroonian volunteers living in a region endemic for *L. loa* as part of a study on *Loa*-associated ophthalmologic, cardiac, and renal impact. Fifty µL of each collected blood sample was examined microscopically, while an additional 50-100 µL was spotted onto filter paper.

### DNA extraction from blood spots

Blood spots were partitioned into 6 mm circular sections (10 µL dried blood each) using disposable sterile biopsy punch tools (Acuderm, Inc., Ft. Lauderdale, FL, USA). A set of 36 punched blood spots (5 punches per sample) were submerged in 200 µL phosphate buffered saline (PBS) and subjected to DNA extraction by the zinc BB/Qiagen spin column method described above. A second set of 36 punched blood spots (2 punches per sample) was transferred into sterile tubes containing 2 ml easyMAG lysis buffer (BioMerieux, Durham, NC, USA), pulse vortexed for 15 seconds, and then incubated for 10 minutes at room temperature. Samples were extracted into 50 µL easyMAG elution buffer according to manufacturer's recommendations for off-board lysis. A third set of 12 punched blood spots (4 punches per sample) was immersed in 200 µL distilled water and boiled for 10–30 minutes at 99°C while shaking.

### Real-time PCR assays

qPCR was performed in an ABI 7900 sequence detection system using Taqman fast chemistry reagents (Applied Biosystems, Carlsbad, CA, USA) and primer/probe sets described in table 1. Amplification conditions were 20 seconds at 95°C followed by 40 cycles of 1 second at 95°C and 20 seconds at 60°C. Quality of template was confirmed for all samples using a control primer/probe set targeting a conserved region of the eukaryotic 18S ribosomal RNA gene (Applied Biosystems, Carlsbad, CA, USA).

### Statistical analysis and calculations

All statistical analyses were performed using GraphPad Prism 5.0 (GraphPad Software, Inc., San Diego, CA, USA). For each qPCR assay of a clinical sample, the number of mf present in the template was extrapolated from a standard curve derived from blood samples spiked with limiting dilutions of mf, using SDS 2.2.2 software (Applied Biosytems, Carlsbad, CA, USA). These extrapolated values were adjusted by the ratio of the percentage of blood spot material used as template (1–10%) compared to the percentage of spiked blood sample used as template (1%) to estimate the number of mf present in each blood spot. The corrected estimates were then divided by the volume of blood processed for DNA extraction (50 µL for boiled samples vs. 20 µL for all other samples) to determine the final estimates of mf concentration for each blood spot sample.

## Results

A screen of our *L. loa* mf-stage cDNA library produced sequence information for 1,882 ESTs, which were assembled into 518 unique contigs by dCAS analysis. From these, 18 potential PCR targets were identified by virtue of having limited similarity to all publicly available nematode ESTs, to the non-redundant protein database (nr), and to clustered ESTs derived from *L. loa* L3 larvae. Each candidate transcript included a start codon and stop codon separated by at least 200 base pairs, indicating a potential ORF (table 2).

**Table 2:** Candidate PCR assay targets based on dCAS bioinformatics analysis of *L. loa* microfilaria EST library screen.

Contig name	Genbank accession number	ORF length (bp)
LLMF3	HM753544	378
LLMF49	HM753550	252
**LLMF72**	**HM753552**	**237**
LLMF99	HM753553	186
LLMF103	HM753536	195
LLMF178	HM753537	219
LLMF188	HM753538	204
LLMF199	HM753539	267
LLMF212	HM753540	201
**LLMF269**	**HM753541**	**387**
LLMF274	HM753542	267
LLMF288	HM753543	219
LLMF342	HM753545	288
LLMF357	HM753546	645
LLMF401	HM753547	615
LLMF415	HM753548	240
LLMF475	HM753549	874
LLMF505	HM753551	392

Contigs selected for further investigation by real-time PCR are shown in bold.

**Figure 1:**
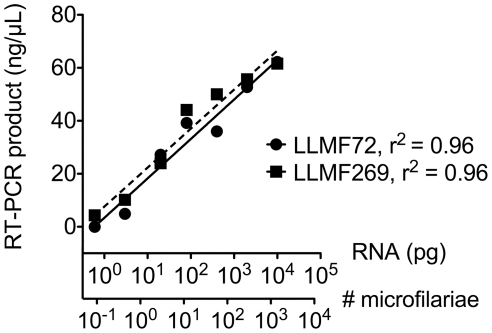
Reverse transcriptase PCR (RT-PCR) of limiting dilutions of *L. loa* total RNA. Shown are concentrations of specific PCR product using primers for targets LLMF72 (circles, solid line) and LLMF269 (squares, dashed line). The lower X-axis indicates the number of *L. loa* microfilariae corresponding to the amount of RNA used as template (upper X-axis). Each point represents the result of a single assay.

Amplification of the 18 candidate transcripts was confirmed by RT-PCR using primers corresponding to the 5′ and 3′ ends of predicted ORFs. All but one of the RT-PCR reactions produced specific PCR products (data not shown). Two targets (predicted ORFs from contigs LLMF72 and LLMF269) were chosen for further evaluation based on abundance of specific RT-PCR product and lack of other nonspecific products. Using limiting dilutions of *L. loa* total RNA as template, RT-PCR detected as little as 3.2 pg (LLMF72) or 0.64 pg (LLMF269) total RNA, corresponding to the RNA present in a fraction of 1 mf (figure 1). For both targets the final concentration of specific PCR product was directly proportional to the log amount of RNA template used.

**Figure 2:**
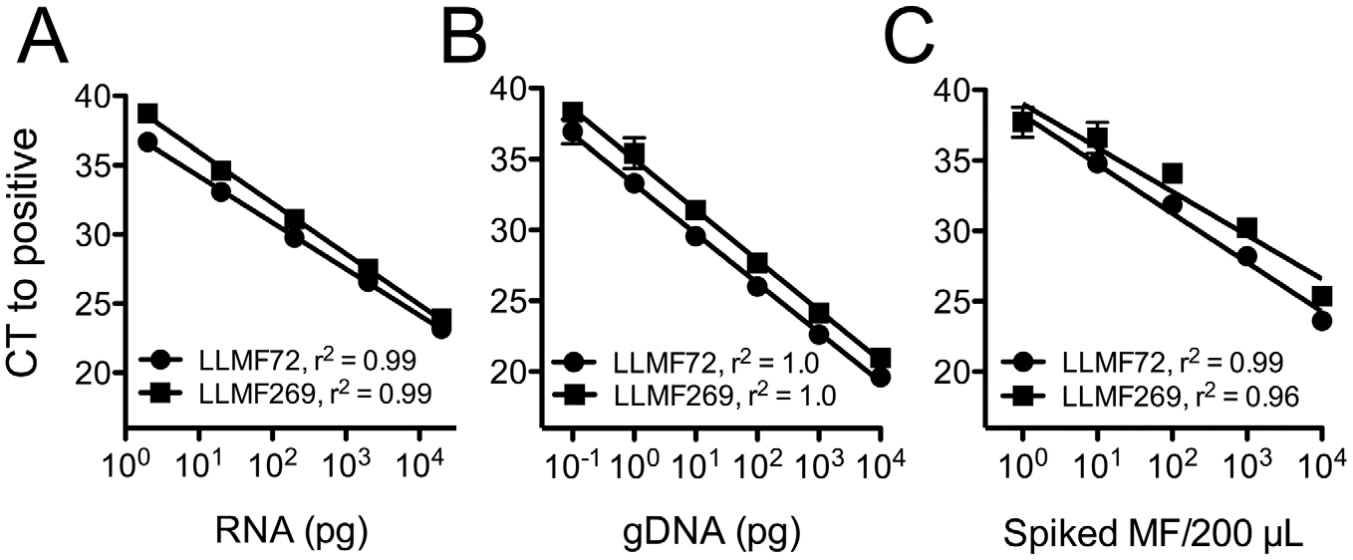
Real-time PCR (qPCR) assays incorporating LLMF72 and LLMF269 targets. Shown are the results of Taqman qPCR assays using primer/probe sets for LLMF72 (circles) and LLMF269 (squares). Each point represents the mean and standard deviation of duplicate assays. Panel A: limiting dilutions of cDNA template created by reverse transcription of *L. loa* total RNA. Panel B: limiting dilutions of highly purified *L. loa* genomic DNA template. Panel C: total DNA extracted from whole blood spiked with limiting dilutions of intact *L. loa* microfilariae.

To shorten the running time of the assay and possibly gain sensitivity, Taqman real-time PCR (qPCR) primers and probes were designed for the LLMF72 and LLMF269 targets. Following reverse transcription, qPCR detected limiting dilutions of *L. loa* total RNA from 20 ng to 2 pg, with a linear relationship between the log amount of RNA used and the number of reaction cycles needed to detect a signal above baseline (figure 2a). Neither qPCR assay detected cDNA prepared from *B. malayi* mf RNA (10-fold dilutions from 20 ng to 2 pg), although from these same *B. malayi* samples a conserved region of the 18S ribosomal RNA gene could be detected using a primer/probe set targeting this sequence (data not shown).

Similar to the situation with total RNA, both qPCR assays detected limiting dilutions of *L. loa* genomic DNA from 10 ng to 0.1 pg. A linear relationship was again observed between the amount of genomic DNA used as template and the number of reaction cycles needed to detect signal above baseline (figure 2b). Neither qPCR assay detected genomic DNA from *B. malayi, O. volvulus, W. bancrofti,* or *M. perstans* (10-fold dilutions from 10 ng to 0.1 pg), although all samples were detected in a linear fashion using the 18S rRNA primer/probe set (data not shown). When both LLMF72 and LLMF269 primer/probe sets were used together in a single assay, there was no reduction compared with the LLMF72 assay alone in the number of reaction cycles at which any amount of DNA was detected (data not shown).

The qPCR assays were next evaluated with DNA extracted from whole blood samples spiked with limiting dilutions of intact *L. loa* mf (1 to 10,000 organisms). Using 1% of the total extracted DNA from each sample as template, there was once more a linear relationship between the log number of mf spiked and the number of reaction cycles needed to detect signal above baseline (figure 2c). The lower limit of detection for both assays was 1% of DNA extracted from a single *L. loa* mf. Combining both LLMF72 and LLMF269 primer/probe sets into a single assay did not increase the sensitivity of the assay beyond that seen with the LLMF72 assay alone (data not shown).

**Figure 3:**
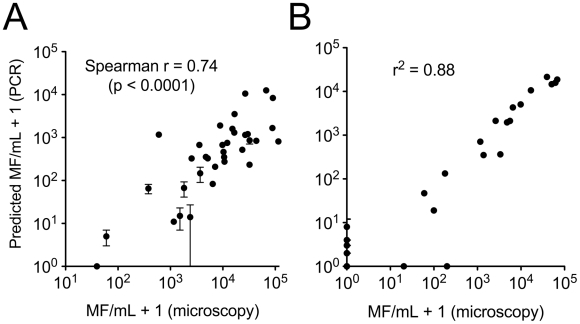
LLMF72 qPCR of total DNA extracted from dried blood spots. Blood was collected from patients living in a *Loa-*endemic area of Cameroon. Each point represents the mean and standard deviation of duplicate assays on the same sample of total DNA extracted from 20 µL dried blood. Panel A: DNA extracted by zinc BB disruption followed by spin column purification. Panel B: DNA extracted by easyMAG. Microscopic quantitation of microfilaremia was performed on 50 µL fresh aliquots at the time of collection.

Recognizing a slight advantage in fewer reaction cycles to positive (i.e., higher sensitivity), the LLMF72 assay was selected for further evaluation with blood samples from a cohort of Cameroonian study subjects with well defined *L. loa* microfilaremia. DNA was extracted from a portion of the dried blood spots using our standard DNA extraction method prior to performing LLMF72 qPCR. Using the spiked blood samples as a standard curve to estimate the concentration of *L. loa* organisms present in each blood spot, there was a significant positive correlation between the extent of microfilaremia predicted by qPCR and the level confirmed previously by microscopy (figure 3a, Spearman r = 0.74; *P*<0.0001). Among 36 blood spots evaluated, only one (40 mf/mL by microscopy) was negative by qPCR.

A second set of 36 blood spots was subjected to automated DNA extraction by easyMAG, and concentration of mf was again estimated by LLMF72 qPCR. Among this set, there was a strong linear correlation between predicted and observed microfilaremia (figure 3b, r^2^ = 0.88; *P*<0.0001). There were two samples positive by microscopy (20 mf/mL and 200 mf/mL) but negative by qPCR. Sixteen of the blood spots in this set were collected from individuals who were apparently amicrofilaremic by microscopy. Five of these samples, however, contained detectable *L. loa* genomic DNA by qPCR (predicted organism burden 1–7 mf/mL).

**Figure 4:**
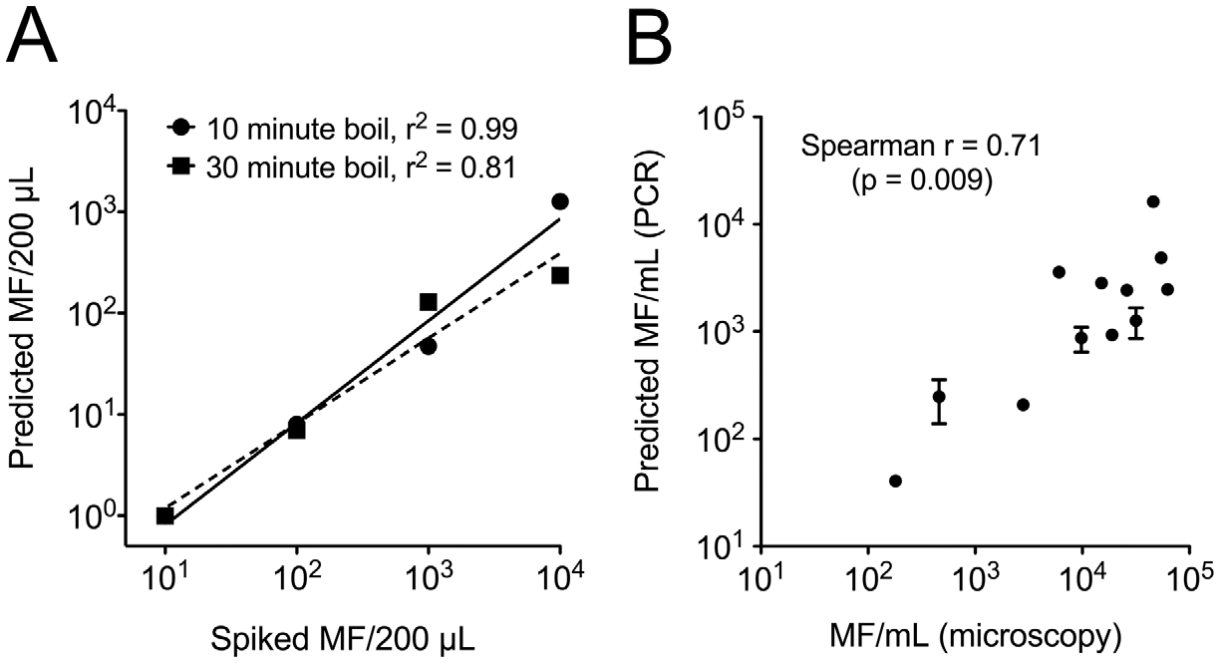
qPCR of boiled blood spots, using the LLMF72 Taqman primer/probe set. Each point represents the mean and standard deviation of duplicate assays. Panel A: Whole blood spiked with limiting dilutions of intact *L. loa* microfilariae boiled for 10 minutes (circles, solid line) or 30 minutes (squares, dashed line). Panel B: total DNA extracted from dried blood spots (40 µL) collected from *L. loa* infected individuals. Microscopic quantitation of microfilaremia was performed on 50 µL fresh aliquots at the time of collection.

To investigate whether time and effort of DNA extraction could be reduced, DNA was extracted from another set of spiked whole blood samples by boiling for 10–30 minutes. Using a standard curve derived from the previously spiked blood samples subjected to zinc BB/spin column extraction, the LLMF72 qPCR assay was positive for all boiled blood samples except the sample spiked with a single organism (figure 4a). There was a linear relationship between number of organisms spiked and number estimated by qPCR, with no increase in DNA extraction efficiency observed with longer boiling time. Centrifugation of boiled samples and use of the supernatants as template did not have any effect on qPCR assay results (data not shown). Efficiency of DNA extraction by boiling was also evaluated with a set of 12 dried blood spots. Boiling resulted in detection of *L. loa* DNA by LLMF72 qPCR in all 12 samples. Furthermore, there was a significant positive correlation between organism burden as assessed by microscopy and organism burden as estimated by qPCR (figure 4b, Spearman r = 0.71; *P* = 0.009).

Overall, our qPCR assay was positive for 65 of 68 samples with parasitologically proven *L. loa* microfilaremia (sensitivity 96%) following various methods of DNA extraction. Performance of qPCR compared to microscopy for each extraction method is summarized in table 3.

**Table 3:** Performance of LLMF72 qPCR assay with blood spots in comparison to microscopy.

DNA extraction method	Microscopy positive		Microscopy negative	
	qPCR positive	qPCR negative	qPCR positive	qPCR negative
Zinc BB and Qiagen column	35	1	ND	ND
easyMAG	18	2	5	11
Boiling	12	0	ND	ND
Total	65	3	5	11

ND: not done.

## Discussion

Improved molecular diagnostics for *L. loa* infection are needed not only in endemic areas where onchocerciasis and lymphatic filariasis elimination efforts are disrupted but also in clinical laboratories of resource-rich countries where the relative infrequency of filarial infections limits the utility of conventional parasitology methods to confirm infection in immigrants and travelers. In this study, we undertook bioinformatics analysis of filarial ESTs to identify candidate targets for *Loa*-specific qPCR assays. From an initial set of 18 candidates, we selected two target sequences (LLMF72 and LLMF269) to develop working real-time PCR assays and defined the performance parameters of one (LLMF72) using blood samples from individuals with known *L. loa* infection status.

Our search for assay targets initially focused on mf-specific transcripts, based on the assumption that such targets would be most abundant in the setting of microfilaremia. Once it became clear that the assay performed equally well with genomic DNA compared with RNA, the advantages of a DNA template (greater stability, no need for extra time and effort of reverse transcription) became readily apparent. Abandoning the transcriptional specificity of an mf-specific RNA target was not a concern, as the mf stage is the only one in which organisms are found in the bloodstream. Furthermore, free DNA probably does not exist in the absence of organisms due to the relative impermeability of the microfilarial sheath. Nonetheless, detection of RNA by real-time PCR following reverse transcription quantitatively assesses expression in human blood and could be applied to estimating transmission potential in vector populations [19], [20] should *L. loa* elimination campaigns be contemplated.

Subsequent to the development of our assays, collaboration with the Broad Institute at Harvard/MIT resulted in sequence assembly and initial annotation of the genomes of *L. loa, O. volvulus*, and *W. bancrofti* (http://www.broadinstitute.org/annotation/genome/filarial_worms/MultiHome.html). Preliminary analysis of the LLMF72 and LLMF269 target sequences indicate that they reside within single-copy genes encoding hypothetical proteins. Both target sequences have similarity to single regions of the *B. malayi*, *W. bancrofti*, *Schistosoma mansoni*, and *Caenorhabditis elegans* genomes, although there is no evidence of gene expression among ESTs of these other organisms. Species specificity of the targets is conferred by a lack of sequence similarity at the primer/probe binding sites. Consequently, our assays are negative with as much as 10 ng of purified genomic DNA (equivalent to 10^4^–10^5^ mf) from *B. malayi, W. bancrofti, O. volvulus,* or *M. perstans*. This level of specificity is extremely important, as *L. loa* may be co-endemic with both *W. bancrofti* and *M. perstans*, whose life cycles also include bloodstream mf.

Perhaps the most notable aspect of our qPCR assays is their sensitivity. We achieved a lower limit of detection equal to 2 pg reverse-transcribed RNA, 0.1 pg genomic DNA, or 1% of DNA extracted from 200 µL of whole blood spiked with a single *L. loa* mf. Using DNA extracted from clinical samples as template, the LLMF72 qPCR assay was able to detect a single mf in a 20 µL dried blood spot (equivalent to a burden of 50 mf/mL). Three false negative results were obtained with blood spot samples where mf burden was very low (20–200 mf/mL, or up to 4 organisms in a 20 µL blood spot). In light of the observed lower limits of detection for purified genomic DNA and spiked whole blood samples, these false negatives were most likely due either to sampling error (no organisms present in the processed blood spot) or to issues with the DNA extraction process.

Previous reports of *L. loa*-specific PCR assays have not included explicit determination of analytic sensitivity for purified DNA or microfilaria burden [8], [12], though assays developed by Tourre *et al* targeting the gene for a 15 kD protein antigen identified individuals with parasitologically proven amicrofilaremic or occult loiasis [9]-[11]. In contrast to qPCR, these conventional PCR assays require significantly longer run times (in particular where nested PCR is used) and also rely on time-consuming gel-based detection methods. Finally, qPCR has the distinct advantage of being quantitative, allowing for estimation of mf burden.

Mf burdens predicted by the LLMF72 qPCR assay were typically 2- to 10-fold lower than microscopic observations, no matter which DNA extraction method was used. These predictions were based on a standard curve derived from spiked whole blood samples, further indicating that extraction from dried blood spots is likely less efficient than extraction from fresh whole blood. Consequently, the qPCR assay will likely be even more sensitive, consistent, and accurate with larger volume (100 µL and above) fresh blood samples. Microfilaremia estimation was most accurate and most consistent with samples subjected to easyMAG extraction. This automated extraction process will therefore be preferred in laboratory settings where such equipment is available.

It is nonetheless encouraging that boiling blood spots for 10 min enabled detection of as few as 180 mf/mL (7 organisms in a 40 µL blood spot). These numbers favor successful detection of microfilaremia well below the threshold for post-ivermectin encephalopathy in endemic areas where conditions may necessitate small sample volumes, delay between sample collection and processing, and simplified extraction of template. Another advantage of using blood spots is that these are already routinely collected for screening purposes (Ov16 rapid diagnostics, filarial-specific antibodies) in areas co-endemic for other filarial infections and would facilitate simultaneous evaluation for multiple infections.

The LLMF72 qPCR assay also detected low-level microfilaremia in 5 of 16 samples thought to be amicrofilaremic by microscopy. These discordances are unlikely attributable to false-positive assay results, because DNA extraction and assay set-up were conducted under rigorous conditions designed to protect against cross-sample contamination, and all qPCR runs included internal no template controls with verified negative results. Rather, the molecular assay is likely detecting small numbers of microorganisms that were missed by microscopy due to sampling error with the 50 µL aliquots that were examined. Previously published qualitative *L. loa* PCR assays have yielded positive results on blood samples from individuals with documented subconjunctival adult worms but no detectable microfilaremia by conventional methods [9], [10], [21]. Among the samples we tested, all five amicrofilaremic but qPCR positive specimens were also positive by serology for IgG against *L. loa* SXP-1 [7], supporting infection with *L. loa*. Corroboration of this interpretation with additional samples would further demonstrate the utility of our molecular assay for detecting low-level microfilaremia in situations where microscopy may suffer from some degree of subjectivity.

Aside from their sensitivity, specificity, and ability to objectively quantify microfilaremia, our qPCR assays offer some advantages compared to conventional parasitology. First, they eliminate the need for specialized training in filarial morphology, thereby making the assay accessible to anyone with general training in PCR techniques. Second, they enable rapid and high throughput sample processing, which would be of great benefit to centralized laboratories conducting population-based screening. Finally, our *L. loa* assay could be multiplexed with quantitative real-time PCR assays for other pathogens (e.g *W. bancrofti* and *M. perstans*), using different fluorescent reporters [22].

Our qPCR assays also have several limitations that must be recognized. Similar to microscopy, optimum PCR performance requires proper timing of blood collection at mid-day when microfilaremia is greatest; however, a microfilaremia kinetics study by Kamgno *et al*. suggests that smaller numbers of organisms may be detectable outside the window of peak microfilaremia [23], and a nomogram may be used to extrapolate qPCR results to predict true parasite burden. Another important issue is that the assays in their current form are still not practical for point-of-care use in endemic areas. One possible solution is to adapt both DNA extraction and real-time PCR processes to be performed on a handheld battery-operated microfluidic device [24]–[27]. Alternatively, the targets could be incorporated into loop-mediated isothermal amplification (LAMP) assays, which are carried out at a single temperature, can use whole blood as template, and can be interpreted using a simple visual color change reporting system [28], [29]. Finally, in considering the possibility of target sequence variability, it will be important to demonstrate preservation of sensitivity with clinical samples from endemic regions outside of southern Cameroon. Notably, PCR positivity has already been demonstrated with blood obtained from patients exposed in Nigeria, Benin, and the Central African Republic (data not shown).

In summary, we have developed a quantitative real-time PCR assay capable of estimating *L. loa* mf burden from small volume blood samples. The assay is highly sensitive, species specific, and may be used for rapid, high-throughput screening either with extracted DNA or with boiled whole blood. The assay is ready for immediate use in clinical laboratories where real-time PCR equipment is available and may eventually be adapted for use in resource-poor endemic areas. This assay represents an important step forward in the diagnosis of loiasis and may ultimately be of benefit in global health campaigns to eliminate filarial diseases.
